# Enteric Bacteria and Cancer Stem Cells

**DOI:** 10.3390/cancers3010285

**Published:** 2011-01-14

**Authors:** Jun Sun

**Affiliations:** 1 Gastroenterology & Hepatology Division, Department of Medicine, University of Rochester, 601 Elmwood Avenue, Rochester, NY 14642, USA; E-Mail: jun_sun@urmc.rochester.edu; Tel.: +10-585-276-3798; Fax: +10-585-275-8118; 2 Department of Microbiology and Immunology, University of Rochester, 601 Elmwood Avenue, Rochester, NY 14642, USA; 3 Wilmot Cancer Center, University of Rochester, 601 Elmwood Avenue, Rochester, NY 14642, USA

**Keywords:** Wnt, JAK-STAT, JNK, inflammation, intestine, stem cell, cancer

## Abstract

Intestinal bacteria can contribute to cell proliferation and cancer development, particularly in chronic infectious diseases in which bacteria and/or bacterial components might interfere with cell function. The number of microbial cells within the gut lumen is estimated to be 100 trillion, which is about 10-times larger than the number of eukaryotic cells in the human body. Because of the complexity of the gut flora, identifying the specific microbial agents related to human diseases remains challenging. Recent studies have demonstrated that the stemness of colon cancer cells is, in part, orchestrated by the microenvironment and is defined by high Wnt activity. In this review article, we will discuss recent progress with respect to intestinal stem cells, cancer stem cells, and the molecular mechanisms of enteric bacteria in the activation of the Wnt pathway. We will also discuss the roles of other pathways, including JAK-STAT, JNK, and Notch, in regulating stem cell niches during bacterial infections using *Drosophila* models. Insights gained from understanding how host-bacterial interaction during inflammation and cancer may serve as a paradigm for understanding the nature of self-renewal signals.

## 1. Intestinal Stem Cells and Bacteria

**Figure 1.**
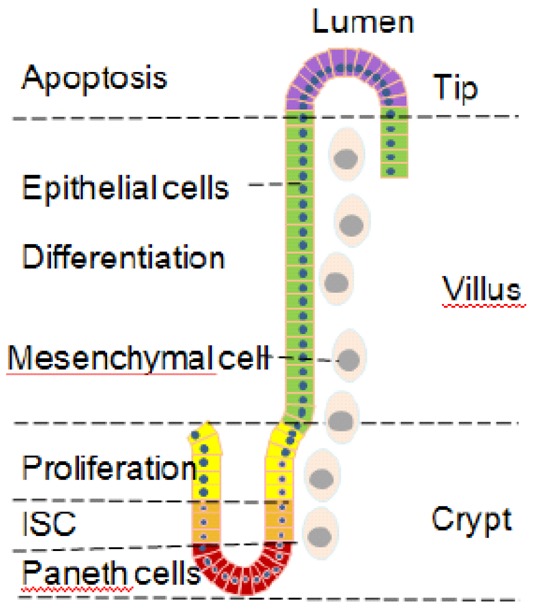
Schematic diagram of the cell population in the mouse small intestinal epithelium. Please note that the intestinal stem cells (ISCs), proliferating progenitor cells, and differentiated cells are located in spatially defined compartments in the intestinal crypt and villus.

Intestinal epithelial cells have barrier, structural, and host defense roles. In the mammalian small intestine, a crypt and villus represents the fundamental repetitive unit. The crypt is composed of 250–300 cells that constantly are actively proliferating, which allows it to generate all the cells required to renew the entire intestinal epithelium in 4–5 days in mice [1]. Intestinal stem cells (ISCs), proliferating progenitor cells, and differentiated cells are located in the intestinal crypt and villus in spatially defined compartments [2,3] (Figure 1). ISCs are located just above Paneth cells or in a permissive microenvironment within the first four to five cell positions from the bottom of the crypt, with some ISCs interspersed among the Paneth cells. Because each crypt is estimated to contain about 30 pluripotent stem cells [3], the intestine is one of the best models for studying adult stem cells *in vivo* [3,4].

Intestinal bacteria modulate important intestinal functions, including nutrient absorption, mucosal barrier fortification, xenobiotic metabolism, angiogenesis, and proliferation [5-7]. More than 500-1000 species of bacteria coexist in the human colon [8,9]. The number of microbial cells within the gut lumen is estimated to be 100 trillion, which is about 10-times larger than the number of eukaryotic cells in the human body [8,10]. Due to the complexity of the gut flora, identifying the specific microbial agents related to stem cells remains challenging.

## 2. Wnt/β-Catenin Pathway

**Figure 2.**
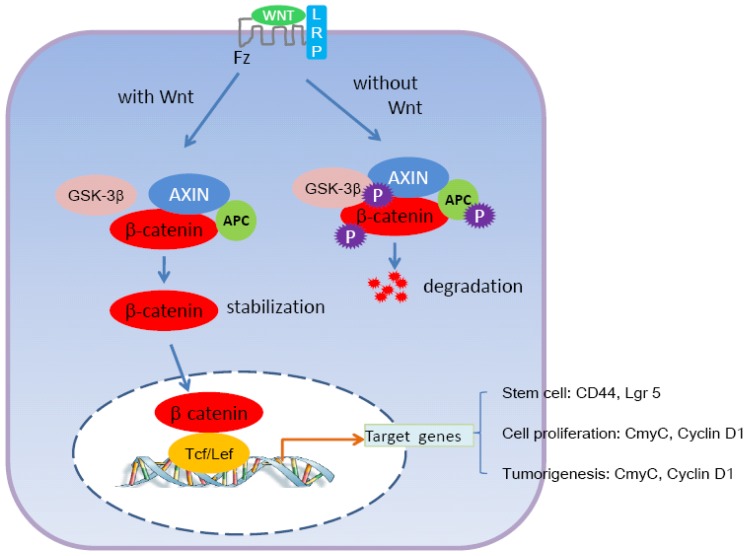
Wnt pathway and its regulation of β-catenin in stem cells and cancer. With Wnt: β-catenin is stabilized and translocated to the nucleus. Nuclear β-catenin binds Tcf, thereby activating Wnt target genes, which in turn regulate stem cells and tumorigenesis. Without Wnt: β-catenin is constitutively degraded.

The Wnt protein is a secreted signaling molecule. In the intestinal epithelium, the Wnt/β-catenin pathway controls a wide variety of normal and pathological processes, including embryogenesis, differentiation, and carcinogenesis [11-13]. The Wnt pathway exists upstream of the β-catenin pathway [14] (Figure 2). Wnt ligands bind to the Frizzled/LRP co-receptor complex and activate the canonical signaling pathway. Axin is recruited to the plasma membrane, resulting in the inactivation of the adenomatous polyposis coli (APC) destruction complex and the subsequent stabilization of β-catenin. When Wnt is activated, β-catenin is stabilized, enabling it to move to the nucleus, where it binds T cell factor (TCF) and thereby triggers the expression of various target genes. Some of these genes, such as leucine-rich repeat-containing G-protein-coupled receptor (Lgr) 5, are involved in stem cell proliferation [15]. APC is a tumor-suppressor protein that is mutated in 80% of human colon cancers. Thus, the activation of Wnt/β-catenin is an early biomarker of colitis-associated colon cancer [16].

Tightly regulated self-renewal mediated by Wnt signaling in stem cells is subverted in cancer cells to allow malignant proliferation [17-22]. Our publications and others have demonstrated that Wnt/β-catenin is a key regulator of intestinal infection and inflammation [4,23-30]. In this review, we intend to discuss the role of Wnt signaling in stem cell division during bacterial infection and colitis-associated colon cancer.

## 3. Janus Kinase and Signal Transducer and Activator (JAK-STAT) Pathway

The JAK-STAT pathway is evolutionarily conserved, from slime molds and worms to mammals. The JAK-STAT system comprises three main components: a receptor, JAK and STAT [31]. The receptor is activated by a signal from interferon, interleukin, various growth factors, or other chemical messengers. This receptor activation triggers the kinase function of JAK, which then phosphorylates the receptor. The STAT protein then binds to the phosphorylated receptor, becomes phosphorylated, and translocates into the cell nucleus, where it binds to DNA and promotes the transcription of target genes that are involved in cell growth, differentiation, and death [31]. In mammals, there are seven *Stat* genes. Disrupted or dysregulated JAK-STAT functionality can result in immune deficiency, inflammation, stem cell defects, and cancer [32,33].

## 4. Bacterial Regulation of Stem Cells in the Gut

### 4.1. Stem Cells in the Infected Drosophila Gut

*Drosophila* is one of the primary model systems for studying the basis of human diseases [34]. In *Drosophila*, the JAK-STAT pathway plays an important role in hematopoiesis, stress response, stem cell proliferation, infection, and antiviral immunity in the intestine [35-39]. A study of *Drosophila* guts infected with the gram-negative bacterium *Erwinia carotovora* revealed that intestinal immune responses and stem cell proliferation are regulated by the Imd and JAK-STAT pathways [40]. Jiang *et al.* and Buchon *et al.* demonstrated that the JAK-STAT signaling pathway enabled intestinal stem cells to maintain tissue homeostasis by increasing their proliferation rate to repair tissue damage caused by infection [40-42]. A genome-wide RNA interference screen also identified genes, including members of the JAK-STAT signaling pathway, involved in the susceptibility or resistance to intestinal infection by the bacterium *Serratia marcescens* [38]. Taken together, these data indicate that the regulation of ISCs by the JAK-STAT pathway is a critical component of host defense against mucosal infection.

Cytoprotective Jun N-terminal kinase (JNK) signaling influences regeneration in the *Drosophila* gut by directing the proliferation of ISCs. A recent study showed that *Pseudomonas aeruginosa* infection activates the JNK pathway, resulting in a dramatic proliferation of ISCs and progenitors to replenish the apoptotic enterocytes induced by infection [43].

Both the JAK-STAT and JNK pathways are required for bacteria-induced stem cell proliferation in *Drosophila* [39,42]. Altered control of gut microbiota in immune-deficient or aged flies correlates with increased epithelial renewal. Hence, epithelial renewal is an essential component of *Drosophila* defense against oral bacterial infection [42].

The increased epithelial renewal upon bacterial infection may be a consequence of the oxidative burst, a major defense mechanism in the *Drosophila* gut. Reactive oxygen species (ROS) participate in signaling for cell proliferation and differentiation via the JAK-STAT and JNK pathways. Oxidative stress is known to regulate both stem and progenitor cells [44]. ROS could also participate in signaling for stem cell activity. The mechanism by which ROS signaling modulates stem cells, however, remains unknown [45], and there have been no reports on the ROS-directed modulation of intestinal stem cell niches during bacterial infection.

Taken together, these studies suggest that infection by *Erwinia carotovora*, *Serratia marcescens*, and *Pseudomonas entomophila*, cell aging, and exposure to xenobiotics can stimulate stem cell activity in the *Drosophila* gut. Gut homeostasis is maintained through a balance between cell damage resulting from the collateral effects of bacterial killing and epithelial repair by stem cell division. The pathways discussed here are conserved during evolution. Thus, some of the processes that are important in flies are also relevant to mammalian host defense. Although the *Drosophila* gut provides a powerful model to study the integration of stress and immunity with pathways associated with stem cell control, mammalian models will provide further insights into the relationship between bacterial infection and intestinal stem cells in the pathogenesis of digestive diseases.

### 4.2. Bacteria Regulate Stem Cells in the Mammalian Intestine

Mammal intestinal homeostasis is achieved by a complex inter-regulation of the immune response, gut microbiota, and stem cell activity. Under the control of the WNT signaling pathway, Lgr5 marks the rapidly dividing cells of the intestinal crypt and identifies a population of cells that is capable of regenerating the entire villous. We recently reported that *Salmonella* infection activates the intestinal stem cell niches through the Wnt/β-catenin pathway and that the bacterial effector protein AvrA is an essential contributor to the activation of intestinal Wnt pathway [46]. *Salmonella* infection is able to alter protein expression and the distribution of stem cell markers. At each of the different stages of bacterial infection, the stem cells exhibit dynamic changes. Although Lgr5 was significantly decreased in the *Salmonella*-infected intestine, cell proliferation was enhanced. The distribution and function of stem cell niches are more complicated in mouse models than those in fruit fly models. The identification of exactly which cells of the intestinal crypt respond to bacterial infection and are involved in epithelial repair will be interesting.

*Helicobacter pylori* frequently colonizes the human stomach, and hosts infected by *cagA*-positive strains are at increased risk of gastric cancer and peptic ulceration. A recent study showed that the bacterial protein CagA was essential for *H. pylori*-induced STAT3 activation [47]. In addition, a DNA microarray analysis using mouse intestinal mucosa showed that *Jak2*, *Stat1* and *Stat3* represented vital genes that were up-regulated four-days post-*Salmonella* infection [48]. The mouse mucosal system, however, is different from the *Drosophila* gut. STAT proteins are intracellular effector molecules of cytokine-modulated signaling in the mammalian immune system [49]. Further research is needed to validate our analysis and determine how JAK-STAT signaling regulates the host response and stem cell proliferation during *Salmonella* infection.

Human mesenchymal stem cells (MSC) participate in the innate immune response against gram-negative bacteria through the secretion of antimicrobial peptides, such as LL-37. Recent *in vivo* studies indicate that MSC may have beneficial effects in the treatment of sepsis induced by bacterial infection [50]. Intestinal Paneth cells secret antimicrobial peptides during bacterial infection. ISCs are located just above Paneth cells, and some may be interspersed among the Paneth cells. Insights into the interaction between Paneth and stem cells could be applied to novel therapeutic strategies to treat infection and inflammation.

## 5. Intestinal Stem Cell-Mediated Tumorigenesis

### 5.1. *Over-Proliferation in the* Drosophila *Gut*

Dysfunctional proliferation in *Drosophila* has been used to mimic the neoplastic transformation in the mammalian gut. Aging is known to activate stem cell activity through PVF2, a PDGF/VEGF-like growth factor [51]. In the normal intestine, JNK signaling and the control of cell proliferation and differentiation by Delta/Notch signaling are carefully balanced to ensure tissue homeostasis [52]. If this balance is lost, then the potential for neoplastic transformation in aging animals increases. In old and stressed intestines, JNK promotes the accumulation of misdifferentiated ISC daughter cells, whereas ectopic Delta/Notch signaling causes ISC abnormal differentiation while also limiting JNK-induced proliferation [52].

Intestinal infection with *Pseudomonas aeruginosa* causes apoptosis of differentiated enterocytes and promotes a dramatic proliferation of ISCs and their progenitors. In *Drosophila* expressing a latent oncogenic form of the *Ras1* oncogene, massive over-proliferation of intestinal cells occurred [43], and the intestines developed excess cell layers that had altered apicobasal polarity reminiscent of dysplasia. These data suggest that infection directly synergizes with the genetic background in predisposed individuals to initiate ISC-mediated tumorigenesis [43].

### 5.2. Cancer Stem Cells in Mammalian Studies

Colon cancer might arise from late progenitors or even an early differentiated cell [53]. Increasing evidence supports the hypothesis that human colon cancer harbors a population of cells with cancer stem cell (CSC) characteristics [54] (Table 1). Both Lgr 5 and CD133 were shown to induce the activation of the Wnt pathway, leading to efficient tumor formation. Lgr 5 is overexpressed not only during the early stages of colorectal tumorigenesis, but also during the late stages [55]. CD133 has also been reported to be a CSC marker [56,57]. However, a recent study found that CD133^+^ cells were not exclusively localized to stem cells, but could be found in the majority of differentiated epithelial cells in organs with hollow ductal and nonductal luminal cavities, including the colon [58]. Although immunostaining with Lgr5 and CD133 identified distinct subpopulations of cells that were in close proximity, these two markers did not co-stain any cells in human intestinal tissues [59]. CD44+/CD166+ cells that do not express CD133 were also identified as a subgroup of human colorectal cancer cells that are uniquely capable of inducing tumors in mice [60]. OLFM4 is a robust marker for stem cells in the human intestine and also marks a subset of colorectal cancer cells [61]. Phosphorylated-β-catenin (552), which is driven by Akt activation, has been identified as a potential intestinal stem cell marker [62]. PTEN is a lipid and protein phosphatase that acts as a negative regulator of PI3K-Akt pathway. Intestinal polyposis is initiated by PTEN-deficient ISCs [62]. Moreover, a recent study demonstrated that phosphoinositide 3-kinase signaling mediates β-catenin activation in intestinal epithelial stem and progenitor cells during intestinal inflammation and colitis-associated colon cancer [63].

### 5.3. Bacterial Regulation of Wnt/β-Catenin Pathways in Mouse Cancer Models

Accumulating evidence suggests that bacterial infections and their effects on undifferentiated and mature enteric epithelial cells have critical roles during the initial stages of intestinal cancer [5,64]. Infection with C. *rodentium* increases epithelial cell proliferation and promotes chemically initiated tumors in the colon through the β-catenin pathway [65,66]. Although, *C. rodentium* is not a human pathogen, the bacterial genes required for infection and presumably for the induction of epithelial cell hyperplasia are also present in related human pathogens. *B. fragilis* toxin triggers β-catenin to localize to the nucleus [67]. We have reported that Salmonella-epithelial interactions influence Wnt/β-catenin signaling [27,46,68]. *H. Pylori* is also known to activate the Wnt/β-catenin pathway [69-71]. These observations indicate that the gut flora may render the host susceptible to oncogenic transformation in the colon by promoting the activation of the Wnt/β-catenin pathway.

A recent study further demonstrated that tumors not only possess great heterogeneity, but that CSC properties within a tumor can be gained or lost depending on the microenvironment [53,72]. A previous study showed that not all cells within an APC-induced tumor have the same level of Wnt pathway activation [73,74]. Taken together, these studies demonstrate the essential role of the microenvironment in tumorigenesis. The intestinal flora represent one environmental factor that contributes to tumorigenesis. To the best of our knowledge, no studies on the effects of intestinal bacteria on CSCs have been reported. An assessment of progenitor cell responses to pathogenic intestinal bacteria could serve as an indicator of a predisposition for intestinal dysplasia in humans.

## 6. Conclusions

**Figure 3.**
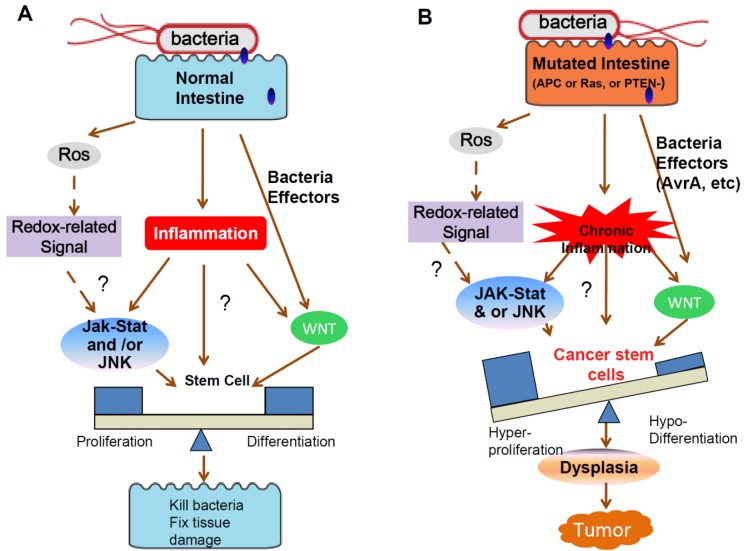
Hypothetical scheme for bacteria-regulated intestinal stem cells and tumorigenesis. (**A**) Enteric bacteria and their products (e.g., the effector protein AvrA) induce ROS and inflammation, leading to the activation of the JAK-STAT, JNK, and/or Wnt pathways. These pathways act as a homeostatic compensatory mechanism to replenish apoptotic enterocytes and replace damaged tissue by stem cell proliferation and differentiation. The stem cell proliferation and differentiation are well-balanced. (**B**) In an intestine containing mutated cells (APC mutation, Ras mutation, or PTEN knockout), the bacteria-induced chronic inflammation and activation of Wnt and JAK-STAT pathways result in an increased number of cell stem cells. Bacterial proteins and products, such as AvrA, participate in the activation of Wnt pathway in the intestine. This uncontrolled inflammation and hypoproliferation then leads to dysplasia and eventually the development of a tumor.

Host-bacteria interactions involve critical signaling pathways, including the Wnt, JAK-STAT, and JNK pathways. Probably due to the nature of gut that requires appropriate wounding repair mechanism, enteric bacteria exploit intestinal stem cell niches through similar pathways in the *Drosophila*, mouse, and human guts. Bacterial infection alters the stem cell niches in the mouse intestine through the activation of the JAK-STAT, JNK, and/or Wnt pathways (Figure 3A), and chronic inflammation, hyperproliferating ISCs, and their progenitors drive cancer initiation, maintenance, and metastasis (Figure 3B). The findings in the *Drosophila* gut provide a framework for understanding intestinal bacterial infections and their effects on undifferentiated and mature enteric epithelial cells during the initial stages of intestinal cancer. An assessment of progenitor cell responses to pathogenic bacteria could serve as an indicator of a predisposition to intestinal dysplasias in humans. Although the*** *Drosophila* gut provides a powerful model to study the integration of stress and immunity with pathways associated with stem cell control, little is known about the relationship between bacterial infection and intestinal stem cells in mammalian models. Therefore, a systematic analysis of whether and how specific environmental factors and the intestinal microbiota contribute to the proliferation of cancer stem cells in the mammalian intestine is important. The insights into the bacterial regulation of intestinal stem cells and cancer stem cells will uncover novel mechanisms of tumorigenesis, and such findings can then be applied to the development of new therapeutic strategies, not only for colon cancer but also for bacterial infections and chronic inflammation.

## Figures and Tables

**Table 1.** A summary for colon cancer stem cell markers.

**Markers**	**Involved pathway(s)**	**Ref.**
Lgr 5	β-catenin, Wnt	[3,55,59,61]
EpCAM, CD44, and CD166	Epithelial cell adhesion	[60]
CD133?	Wnt	Support CD133: [56,57] Does not support CD133: [58]
OLFM4	Wnt	[61]
Phosphorylated-β-catenin (552)	Phosphoinositide 3-kinase, Akt, PTEN, β-catenin, Wnt	[62,63].
